# Comparative transcriptome provides insights into the selection adaptation between wild and farmed foxes

**DOI:** 10.1002/ece3.8071

**Published:** 2021-08-30

**Authors:** Xiufeng Yang, Guangshuai Liu, Qi Wang, Xiaodong Gao, Tian Xia, Chao Zhao, Huashan Dou, Honghai Zhang

**Affiliations:** ^1^ College of Life Science Qufu Normal University Qufu China; ^2^ Hulunbuir Academy of Inland Lakes in Northern Cold & Arid Areas Hulunbuir China

**Keywords:** farmed fox, homologous gene, human domestication, positive selection, transcriptome

## Abstract

The silver fox and blue fox are economically important fur species and were domesticated by humans from their wild counterparts, the arctic fox and red fox, respectively. Farmed foxes show obvious differences from their wild counterparts, including differences in physiology, body size, energy metabolism, and immunity. However, the molecular mechanisms underlying these differences are presently unclear. In this study, we built transcriptome libraries from multiple pooled tissues for each species of farmed fox, used RNA‐seq to obtain a comprehensive dataset, and performed selection analysis and sequence‐level analyses of orthologous genes to identify the genes that may be influenced by human domestication. More than 153.3, 248.0, 81.6, and 65.8 million clean reads were obtained and assembled into a total of 118,577, 401,520, 79,900, and 186,988 unigenes with an average length range from 521 to 667 bp for AF, BF, RF, and SF, respectively. Selective pressure analysis showed that 11 and 14 positively selected genes were identified, respectively, in the two groups (AF vs. BF and RF vs. SF). Several of these genes were associated with natural immunity (*CFI* and *LRRFIP1*), protein synthesis (*GOLGA4*, *CEP19* and *SLC35A2*), and DNA damage repair (*MDC1*). Further functional enrichment analyses demonstrated that two positively selected genes (*ACO1* and *ACAD10*) were involved in metabolic process (GO:0008152, *p*‐value = .032), representing a significant enrichment. Sequence analysis of 117 orthologous genes shared by the two groups showed that the *LEMD2*, *RRBP1,* and *IGBP1* genes might be affected by artificial selection in farmed foxes, with mutation sites located within sequences that are otherwise highly conserved across most mammals. Our results provide a valuable transcriptomic resource for future genetic studies and improvement in the assisted breeding of foxes and other farmed animals.

## Graphical abstract

The silver fox and blue fox are economically important fur species and were domesticated by humans from their wild counterparts, arctic fox and red fox, respectively, over a few centuries. Farmed foxes show obvious differences from their wild counterparts, including differences in physiology, body size, energy metabolism, and immunity. However, the molecular mechanisms underlying these differences are presently unclear. Our results provide a valuable transcriptome resource for future genetic studies and improvement in the assisted breeding of fox and other farmed animals.

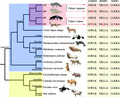

## 1 INTRODUCTION

There is a long history of domestication of wild animals. Humans choose animals with certain characteristics for breeding to satisfy their needs (Li et al., 2020; Rimbault & Ostrander, 2012), and this evolutionary process, during which organisms adapt to living closely with humans, is accompanied by pronounced morphological, physiological, and behavioral changes (Sato et al., 2020). The domestication characteristics of many species have been accumulated and continuously maintained over years of selection, and the related molecular mechanisms have been studied with an abundance of genomic and transcriptomic data resources (Cui et al., 2014; Jiang et al., 2015). As a part of human civilization, the exploration of the genetic mechanism of animal domestication could be used not only to further select traits that are useful to humans but also to improve domestication efficiency (Peng et al., 2014; Song et al., 2019). To date, numerous studies have explored the molecular mechanisms involved in domestication and genetic improvements in several domesticated animals, such as dogs, sheep, cattle, rabbits, and mink (Alberto et al., 2018; Daetwyler et al., 2014; Li et al., 2020; Morris et al., 2020; Sato et al., 2020).

*Vulpes* is one of the world's most widely naturally distributed groups of terrestrial carnivores, and members of this genus play important roles in maintaining ecosystems (IUCN, 2018). They occupy a wide variety of ecosystems, including deserts, grasslands, forests and agricultural and human‐dominated environments (Statham et al., 2014). Two *Vulpes* species were domesticated 270 years ago in Canada: the blue fox, a variant of the arctic fox, and the silver fox, a variant of the red fox (Trut, 1999, 2001). Foxes are important fur species that are widely raised around the world, and the fox fur trade has become one of the three pillar industries of the world fur industry (Zhou, 1987). At present, research on these two farmed fox species focuses mainly on their physiology, biochemistry, breeding, and reproduction (Cao et al., 2019; Zhong et al., 2019). Recently, a genomic study of farmed silver fox focused on the genetic mechanisms underlying behavioral differences identified a few strong positional candidate genes for tame, aggressive, and conventional behavior (Kukekova et al., 2018). Under long‐term human management, wild and farmed foxes have major differences in behaviors (Zhang, 2004), body size, and feeding habits (Kukekova et al., 2008, 2011). However, the molecular mechanisms of these differences remain unclear.

Developments in sequencing technology provide an opportunity to reveal the genetic mechanism underlying the rapid behavioral evolution that occurs during domestication. Transcriptome analysis is an economic strategy to assemble a large number of protein‐coding genes in nonmodel organisms for which genome sequences are not yet available and can be used to identify adaptive genetic mechanisms. Earlier, to characterize the genetic mechanism of differences between wild and domestic animals, comparative transcriptome studies have been performed on a wide range of organisms, such as dogs and wolves, wild and domestic rabbits, and wild and domestic silkworms (Fang et al., 2015; Sato et al., 2020; Yang et al., 2018). Here, we sequenced the pooled transcriptome of multiple tissues of three blue foxes and one silver fox and compared them with those of their wild counterparts to explore genes that might be influenced by domestication and associated with morphological and physiological differences. Furthermore, we aimed to identify gene‐associated simple sequence repeats (SSRs) to provide new markers for the future construction of high‐resolution genetic linkage maps and reliable genetic markers for the selection of superior fox varieties.

## 2 MATERIALS AND METHODS

### 2.1 Sample collection

Farmed foxes, including one silver fox (SF) and three blue foxes (BF1, BF2, and BF3), were collected from the Xingzeyu fox breeding base in Cangzhou city, Hebei Province, China. All of the selected individuals were typical of the breeds and were unrelated. Kidney, brain, and liver tissue was soaked in RNA stabilization reagent (RNAlater, QI‐AGEN, USA) and stored at 80℃ in ultradeep‐freeze equipment.

The wild fox sequences used in this study included data from one *Vulpes vulpes* (red fox, named RF) and two *Vulpes lagopus* (arctic fox, named AF1 and AF2) originally from Kronoby, Finland. These data were downloaded from the National Center for Biotechnology Information (NCBI) Sequence Read Archive (SRA) database with accession numbers ERR687855, ERR687853, and ERR687854 (Kumar et al., 2015). These data were obtained by sequencing a mixture of brain, liver, and kidney tissue. The arctic fox and blue fox groups were referred to as AF versus BF, and the red fox and silver fox groups were referred to as RF versus SF.

### 2.2 RNA extraction, library construction, and transcriptome sequencing

Total RNA was extracted using an RNeasy Mini Kit (QIAGEN, USA) according to the manufacturer's protocol. Total RNA was monitored for quality on 1% agarose gels, and its purity was checked using a NanoPhotometer^®^ spectrophotometer (IMPLEN, USA). The concentration and integrity of RNA were measured and assessed using the Qubit^®^ RNA Assay Kit (Invitrogen, USA) in Qubit^®^ 2.0 Fluorometer (Life Technologies, USA) and the RNA Nano 6000 Assay Kit of the Agilent Bioanalyzer 2100 system (Agilent Technologies, USA), respectively.

RNA extracts from different tissues (liver, brain, and kidney) of the same individual were pooled. Total RNA (3 μg per individual) was used to construct sequencing libraries with the NEBNext^®^ Ultra™ RNA Library Prep Kit for Illumina^®^ (NEB, USA) following the manufacturer's protocol. The library quality was assessed on the Agilent Bioanalyzer 2100 system, and the libraries were sequenced on the Illumina HiSeq 2500 platform; 125 bp paired‐end reads were generated.

### 2.3 Transcriptome assembly, CDS identification, and gene functional annotation

Raw reads in FASTQ format (Cock et al., 2010) were first filtered for adapter or Poly‐N and low‐quality reads using in‐house Perl scripts to obtain clean reads. The error rate (Q20 and Q30 scores) and GC‐content of the clean reads were calculated at the same time, and the subsequent analysis was based on high‐quality clean reads (Hansen et al., 2010; Jiang et al., 2011). Reads from the three BF samples were combined for transcriptome assembly. High‐quality clean reads were de novo assembled by Trinity 2.9.0 (Grabherr et al., 2011) with min_kmer_cov set to 2 to obtain transcripts. After assembly, the longest transcript sequence from each locus was taken and defined as a unigene, and further analysis was based on these unigenes. The CDS of each putative unigene was extracted using two steps: First, unigenes were subjected to BLAST search in the NR and SwissProt databases with a cutoff of 1e‐5. Second, Estscan (Iseli et al., 1999) software was used to identify the unigenes that did not produce any alignment results and to translate the resultant CDSs into amino acid sequences (Wang et al., 2011). Gene function was annotated based on seven databases (Nt, Nr, KEGG, SwissProt, PFAM, GO, and KOG/COG) to understand the function of transcriptionally expressed genes in detail. The website and parameters of the seven databases are listed Table S1 (Altschul et al., 1997; Finn et al., 2008; Stefan et al., 2008).

### 2.4 Simple sequence repeat detection

MISA (Miller et al., 2012) was used to identify SSRs in the transcriptome. To verify the SSRs, Primer 3 (http://primer3.sourceforge.net/releases.php) was used to design primers for each SSR in batches. Three pairs of primers of length 18–22 were designed for each site, and the length of the amplified target fragment was 100–400 bp. Primers were randomly selected from the SSR of 2/3/4/5/6 nucleotide repeats of BF and SF for PCR amplification and electrophoretic imaging verification. Detailed information regarding the PCR system and amplification conditions can be found in the attachment file of Table S2.

### 2.5 Identification of gene orthologous groups and calculation of Ka/Ks

OrthoMCL (Li, 2003) software was used to construct orthologous groups from the resultant CDS using BLAST‐based approach with dog (*Canis lupus familiaris*, cfa) and giant panda (*Ailuropoda melanoleuca*, aml) downloaded from NCBI as the internal and external reference genomes, respectively. Three methods, including DAVID (Dennis et al., 2003), KOBAS (Xie et al., 2011), and the R package ClusterProfiler (Yu et al., 2012), were used for GO and KEGG enrichment analysis. Muscle 3.8 (Edgar, 2004) was used for sequence alignment and for optimizing the protein alignment results. PAUP 3.0 (Guindon et al., 2005) was used to construct evolutionary trees using the sequence alignment results by Muscle.

In genetics, the value of ω (Ka/Ks) can be used as an indicator of the selective pressure acting on a protein‐coding gene. The value of ω is the ratio of the number of nonsynonymous substitutions per nonsynonymous site (Ka) to the number of synonymous substitutions per synonymous site (Ks). Sites with ω > 1 are usually said to be evolving under positive selection, and those with ω < 1 are usually said to be under purifying selection. The Paml 4.7 (Yang, 2007) package was used to identify orthologous genes that showed a significantly higher Ka/Ks ratio in the wild and farmed groups.

### 2.6 Identification of specific mutations in genes of farmed foxes

In the last section of this article, we focus on the orthologous genes shared by two groups (AF vs. BF and RF vs. SF) to identify the gene mutations specific to farmed foxes. First, we performed specific mutation analysis to identify genes in which the two farmed species had the same amino acid mutation relative to wild species. Then, considering that different famed species may have different strategies to adapt to the artificial breeding environment, we also screened for genes with specific mutations in each of the two farmed species. On this basis, to better understand the conservation of these loci at the mammalian level, we increased the number of target species in the analysis.

## 3 RESULTS

### 3.1 Overview of transcriptome sequencing data

Four transcriptome libraries (SF, BF1, BF2, and BF3) were generated from pooled RNA extracts from different tissues (liver, brain, and kidney) of one SF and three BF. After filtering the raw data, a total of 313.8 million clean reads were remained for further transcriptomic assembly (Table 1). The raw data of RF, AF1, and AF2 were downloaded from the SRA database, and a total of 234.9 million clean reads were remained for further transcriptomic assembly after filtering.

**TABLE 1:** Summary of the sequencing results

Sample	Raw reads	Clean reads	Clean bases (Gb)	Error (%)	Q20 (%)	Q30 (%)	GC (%)
RF^a^	87,641,852	81,623,324	7.67	0.08	93.82	84.17	50.21
AF1^a^	72,243,730	67,227,516	6.32	0.09	93.45	83.75	50.39
AF2^a^	92,135,172	86,128,572	8.10	0.08	93.95	84.33	49.84
SF	69,100,036	65,776,886	9.87	0.01	97.51	93.76	51.31
BF1	88,305,502	83,722,694	12.56	0.01	97.50	93.77	52.19
BF2	94,358,648	90,610,456	13.59	0.02	96.82	92.38	51.38
BF3	77,995,962	73,710,874	11.06	0.01	97.51	93.80	51.28

Abbreviations: AF, Arctic fox; BF, Blue fox; RF, Red fox; SF, Silver fox.

^a^ The data downloaded from the SRA database. Error: sequencing error rate. Q20/Q30: percentage of bases with a Phred value of at least 20/30. GC: The content of G and C.

### 3.2 De novo assembly and functional annotation

Three BF individuals were used to generate a pooled assembly, which we referred to as BF. Two AF individuals were used to generate a pooled assembly, which we referred to as AF. Statistics related to the de novo assemblies of each of the four samples are shown in Table 2. The numbers of unigenes for the four transcriptomic assemblies ranged from 79,900 (RF) to 401,520 (BF). The mean unigene length was between 521 bp (BF) and 667 bp (RF), the N50 lengths ranged from 625 (BF) to 1,205 bp (AF), and the N90 lengths ranged from 240 (AF) to 252 (RF). All these assemblies together generated 786,985 unigenes with an average length of 566 bp. The size distribution and percentage of these transcripts and unigenes are shown in Figure S1 and Table S3.

**TABLE 2:** Statistics of the transcriptome assemblies

Group	Assembly statistics		Total Nucleotides	Total Number	N50	N90	Mean Length	Median Length
AF versus BF	AF	Transcript	110,397,020	142,357	1,759	261	775	324
		Unigene	74,566,356	118,577	1,205	240	629	297
	BF	Transcript	306,057,491	462,192	1,133	256	662	322
		Unigene	209,148,339	401,520	625	241	521	303
RF versus SF	RF	Transcript	76,791,137	97,589	1,533	277	787	368
		Unigene	53,304,583	79,900	1,195	252	667	329
	SF	Transcript	163,288,740	219,562	1,628	260	744	323
		Unigene	107,231,995	186,988	860	241	573	295

Note

N50/N90: The transcript obtained by splicing was arranged from long to short and then accumulated. When the cumulative length>=50%/90% of the total length, then the transcript length is considered N50/N90.

All the unigenes were then searched against seven public databases (Nr, Nt, GO, PFAM, KOG, SwissProt, and KO) using the BLASTX program for annotation. For these datasets, the number of unigenes annotated in different databases and their percentages were calculated separately (Table S4). A total of 157,643, 66,782, 89,599, and 56,919 unigenes were annotated in AF, BF, RF, and SF respectively, and the Nt database (AF: 143,994, BF: 64,151, RF: 84,382, SF: 55,600) contributed the largest number of matches (Figure S2). The percentage of unigenes annotated in at least one database ranged from 39.26% (BF) to 71.23% (RF). Information about the E‐value distribution, similarity distribution, and species distributions in these BLAST analyses is displayed in Table S5 and Figure S3. Notably, the *E*‐value distribution of the top hits in the NR database showed that the percentage of all unigenes yielding a match with an e‐value <10–10 in comparison with the Nr database ranged from 84.4% (AF: 29,814) to 95.0% (SF and RF: 26,468), and the percentage with a match e‐value <10–100 ranged from 35.6% (AF: 12,569) to 43.6% (BF: 11,845). This high similarity suggests that our sequencing and assembly is accurate.

After coding sequence (CDS) BLASTX and unigene prediction, a total of 405,560 (AF: 69,445, BF: 189,908, RF: 51,652, SF: 94,555) CDSs were found, with a minimum length of 51 bp (SF) and a maximum length of 24,885 bp (SF) among the four transcriptomic assemblies (Table 3, Figure S4). Only CDSs longer than 300 nucleotides (130,431 in total; AF: 25,639, BF: 49,966, RF: 23,298, SF: 31,528) were retained and used for future analysis.

**TABLE 3:** Results of CDS BLASTX and prediction

Group	Sample	Number of Blast to Protein database		Number of prediction by Estscan	
		Total	>300	Total	>300
AF versus BF	AF	15,347	11,945	54,098	13,694
	BF	21,250	14,352	168,658	35,614
RF versus SF	RF	13,354	10,258	38,298	13,040
	SF	17,972	13,676	77,472	17,852

Note

>300, Number of CDS longer than 300 nucleotides.

### 3.3 SSR detection and primer design

**FIGURE 1:**
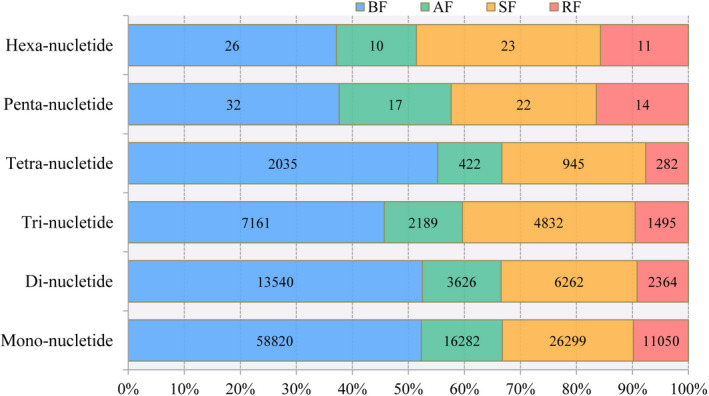
Bar plot of different types of identified SSRs. The y‐axis shows the type of SSRs. The number on the box represents the number of SSRs identified in different fox transcriptomes. Different colors represent different fox species

A total of 157,759 (AF: 22,546, BF: 81,614, RF: 15,216, SF: 38,383) potential microsatellite sites were detected on 125,736 sequences using MISA. In total, 23,773 (AF: 3,698, BF: 11,543, RF: 2,367, SF: 6,165) sequences contained more than 1 potential SSR site, and 6,830 compound (AF: 1,018, BF: 3,331, RF: 654, SF: 1,827) SSRs were present. Detailed results for the SSRs detected in this study are shown in Figure 1 and Table S6.

We then used Primer 3.0 to design three primers for each SSR, and this design process was successful for a total of 59,010 (AF: 9,905, BF: 27,471, RF: 6,547, SF: 15,087) SSRs. We randomly selected two pairs of primers to amplify and agarose gel electrophoresis from Di‐, Tri‐, Tetra‐, Penta‐ and Hexa‐nucleotide SSRs, and the results showed 90% and 50% success rates in BF and SF, respectively (Table S7).

### 3.4 Identification of gene orthologous groups and phylogenomic analyses

We identified a total of 14,750 homologous genes in AF versus BF and 14,443 homologous genes in RF versus SF. To ensure the accuracy of the results, only orthologous genes were selected for the following analysis. After filtering, 812 (AF vs. BF) and 710 (RF vs. SF) single‐copy genes were retained. We then annotated these single‐copy genes according to the GO and KEGG databases to determine their functions.

**FIGURE 2:**
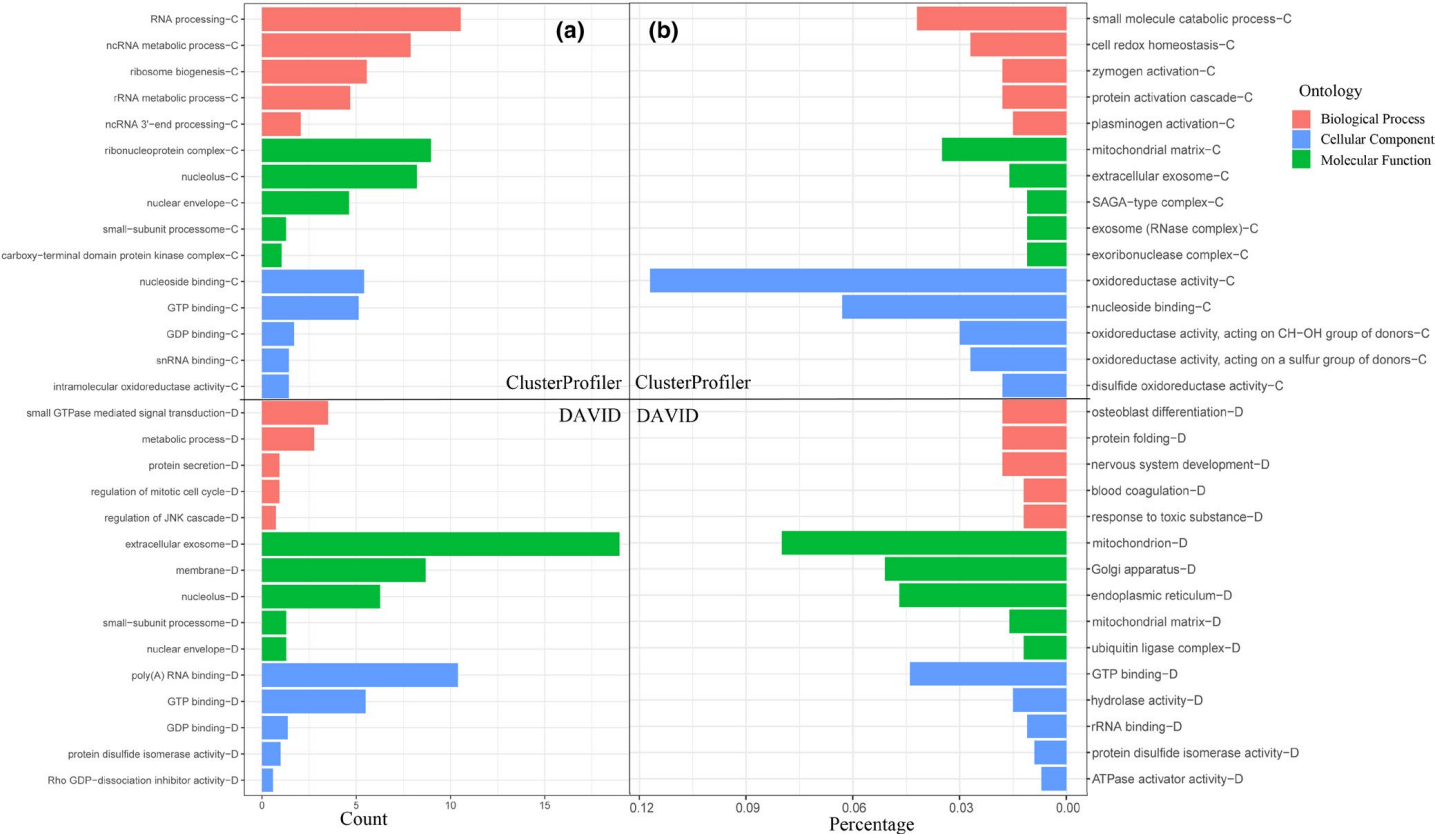
Bar plot of selected GO functional classifications for orthologous genes in (a) AF versus BF and (b) RF versus SF annotated using the ClusterProfiler (–C) and DAVID (‐D) methods. The x‐axis shows the number/percentage of orthologous genes, and the y‐axis shows the enriched GO terms

Different algorithms have been applied in research on the function of orthologous genes, and we used three methods for GO and KEGG enrichment analysis. Ultimately, 1,522 genes in the two groups were annotated with 1,084 GO terms and 37 KEGG pathways (Table 4). Several immune system terms were identified, such as T‐cell receptor signaling pathway (GO:0050852, *p* = .030), immune response‐activating cell surface receptor signaling pathway (GO:0002429, *p* = .046), positive regulation of macroautophagy (GO:0016239, *p* = .053) Regulation of autophagy (cfa04140, *p* = .026). In addition, some genes were annotated with protein‐related pathways, such as negative regulation of protein catabolic process (GO:0042177, *p* = .025), positive regulation of protein catabolic process (GO:0045732, *p* = .018), protein catabolic process (GO:0030163, *p* = .026), protein folding (GO:0006457, *p* = .017), regulation of protein catabolic process (GO:0042176, *p* = .0096), and protein processing in endoplasmic reticulum (cfa04141, *p* = .0074) (Figure 2). Interestingly, we found that eight genes, *F9*, *SERPINE2*, *C1GALT1C1*, *RHOA*, *BIN3*, *APOH*, *FGB,* and *AQP1*, were annotated to Response to wounding (GO:0009611, *p* = .033) in AF versus BF, and six genes, *APOH*, *FGA*, *FGB*, *SERPINC1*, *SERPINE1,* and *SERPINE2*, were annotated in regulation of response to wounding (GO:1903034, *p* = .0497) and negative regulation of response to wounding (GO:1903035, *p* = .0072) in RF versus SF. Cluster analysis showed that the orthologous gene set constructed in this study could satisfactorily cover the genes related to phenotypic differences.

**TABLE 4:** Partial result of GO and KEGG enrichment analyses

ID	GO term	Gene Name	*p*	Group
0009611	Response to wounding	*F9, SERPINE2, C1GALT1C1, RHOA, BIN3, APOH, FGB, AQP1*	.033	AF versus. BF
0010165	Response to X‐ray	*GATA3, CYP27B1F9*	.024	
0043627	Response to estrogen	*XRCC6, GATA3*	.049	
0050852	T‐cell receptor signaling pathway	*BCL10, EIF2B1, GATA3, RBCK1, ZC3H12A*	.030	
0002429	Immune response‐activating cell surface receptor signaling pathway	*BCL10, EIF2B1, GATA3, MNDA, RBCK1, ZC3H12A*	.046	
0006457	Protein folding	*TMX1, TXNDC11, CCT4, VBP1, PPIL4, PDIA6, PDIA5, CCT3, CALR, QSOX1*	.017	
cfa04141	Protein processing in endoplasmic reticulum	*DERL2, TRAF2, PDIA6, RRBP1, EIF2AK1, ATF6, SEC63, CALR, FBXO2, DNAJC3, UBE2J1, DNAJB2, EDEM1,*	.0074	
1903035	Negative regulation of response to wounding	*APOH, FGA, FGB, SERPINE1, SERPINE2*	.0072	RF versus. SF
1903034	Regulation of response to wounding	*APOH, FGA, FGB, SERPINC1, SERPINE1, SERPINE2*	.0497	
0016239	Positive regulation of macroautophagy	*SQSTM1, ULK1, EPM2A, PAFAH1B2, SIRT1*	.0053	
0042177	negative regulation of protein catabolic process	*EFNA1, F13B, PDCL3, SCO1, SERPINE2, UBAC2*	.025	
0045732	positive regulation of protein catabolic process	*GPC3, FOXO1, SEC22B, C4BPB, MYLIP, GGA3*	.018	
0042176	regulation of protein catabolic process	*BAG2, CLU, EFNA1, F13B, FBXL5, GGA3, GPC3, PDCL3, PSMD2, UBAC2, RNF144B, SCO1, SERPINE2, TMEM259,*	.0096	
0030163	Protein catabolic process	*ABHD13, BAG2, CLU, CTSB, CTSD, EFNA1, F13B, FBXL5, FBXO7, GGA3, GPC3, LGMN, PDCL3, PPT1, PSMB2, PSMD2, RBCK1, RIPK1, RNF144B, SCO1, SERPINE2, SIRT1, TMEM259, TSG101, UBAC2, UBE2J2*	.026	
cfa04140	Regulation of autophagy	*ULK2, PIK3C3, ULK1, ATG4A*	.0074	

**FIGURE 3:**
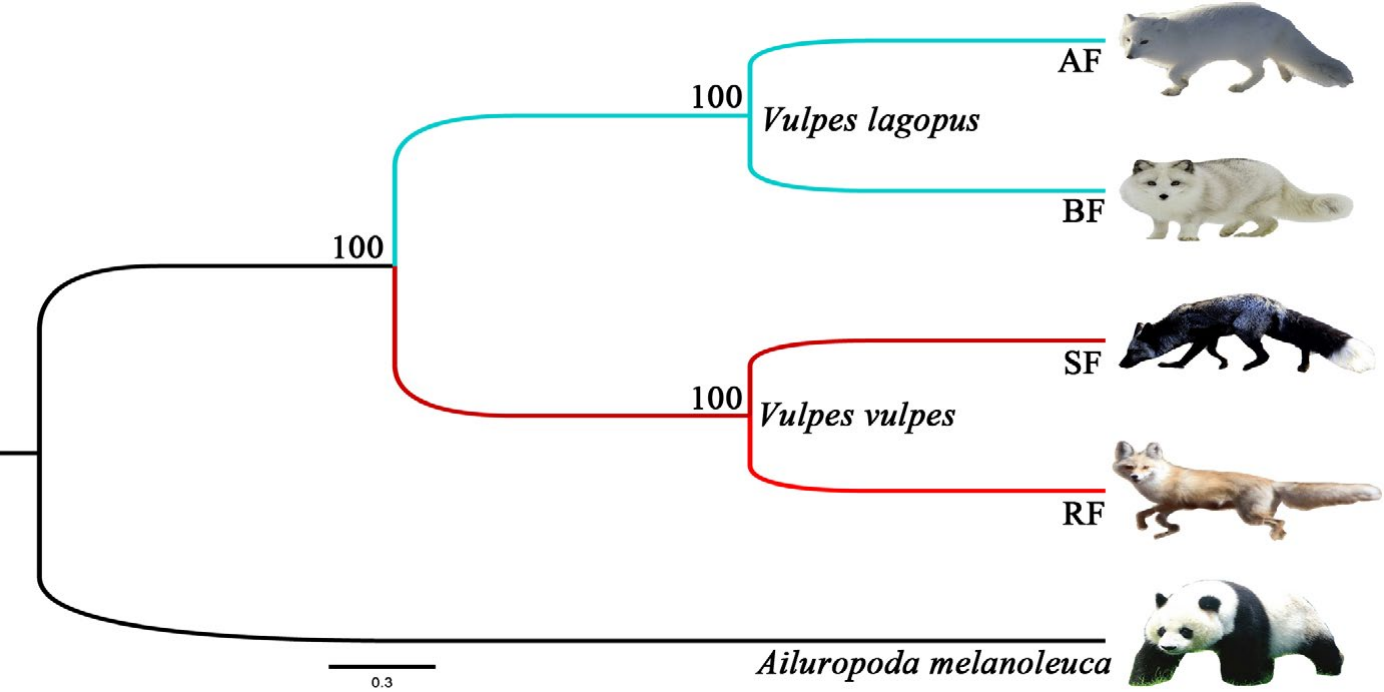
Phylogenetic tree of four species of *Vulpes*. The number along the branch indicates the bootstrap value. *Ailuropoda melanoleuca* was used as an outgroup. AF: arctic fox. BF: blue fox. SF: silver fox. RF: red fox

In total, 171 single‐copy orthologous genes shared by two groups (AF vs. BF & RF vs. SF) were aligned and then used to construct evolutionary trees in PAUP (Figure 3).

### 3.5 Calculation of Ka/Ks to Identify Positively Selected Genes

Next, we performed selective stress analysis on the identified single‐copy orthologous genes with the goal of revealing the genetic mechanism of phenotypic adaptation in response to domestication. The identified 812 (AF vs. BF) and 710 (RF vs. SF) single‐copy orthologous genes were used to calculate the Ka/Ks ratio. Of these, 11 (AF vs. BF) and 14 (RF vs. SF) candidate orthologous genes were identified to be under positive selection with ω > 1 (Table 5).

**TABLE 5:** Positive selection gene

Gene ID	Gene name	Ka/Ks	Group
*GOLGA4*	Golgin A4	1.00051	AF versus BF
*C11orf68*	Chromosome 11 open reading frame 68	1.01649	
*SLC35A2*	Solute carrier family 35 member A2	1.06504	
*FGFBP1*	Fibroblast growth factor binding protein 1	1.17262	
*ADCK2*	AarF domain‐containing kinase 2	1.25811	
*ACAD10*	Acyl‐CoA dehydrogenase family member 10	1.32383	
*CASP3*	Caspase 3	1.37442	
*ACO1*	Aconitase 1	1.3868	
*GRAMD3*	GRAM domain containing 3	1.54881	
*CFI*	Complement factor I	8.47086	
*SLX4IP*	SLX4 interacting protein	19.53611	
*DBN1*	Drebrin 1	1.037	RF versus SF
*SNX15*	Sorting nexin 15	1.03782	
*RPL4*	Ribosomal protein L4	1.05145	
*HP*	Haptoglobin	1.07955	
*IKBKAP*	Inhibitor of kappa light polypeptide gene enhancer in B cells, kinase complex‐associated protein	1.14748	
*HDHD3*	Haloacid dehalogenase like hydrolase domain containing 3	1.17992	
*LRRFIP1*	LRR binding FLII interacting protein 1	1.46571	
*MDC1*	Mediator of DNAdamage checkpoint 1	1.49301	
*TMEM41B*	Transmembrane protein 41B	1.92442	
*AOPEP*	Aminopeptidase O	1.93112	
*CEP19*	Centrosomal protein 19	2.10934	
*MCM7*	Minichromosome maintenance complex Component 7	7.25294	
*C1ORF74*	Chromosome 1 open reading frame 74	9.22315	
*TMEM132A*	Transmembrane protein 132A	11.21415	

### 3.6 Identification of specific mutations in genes of farmed foxes

**FIGURE 4:**
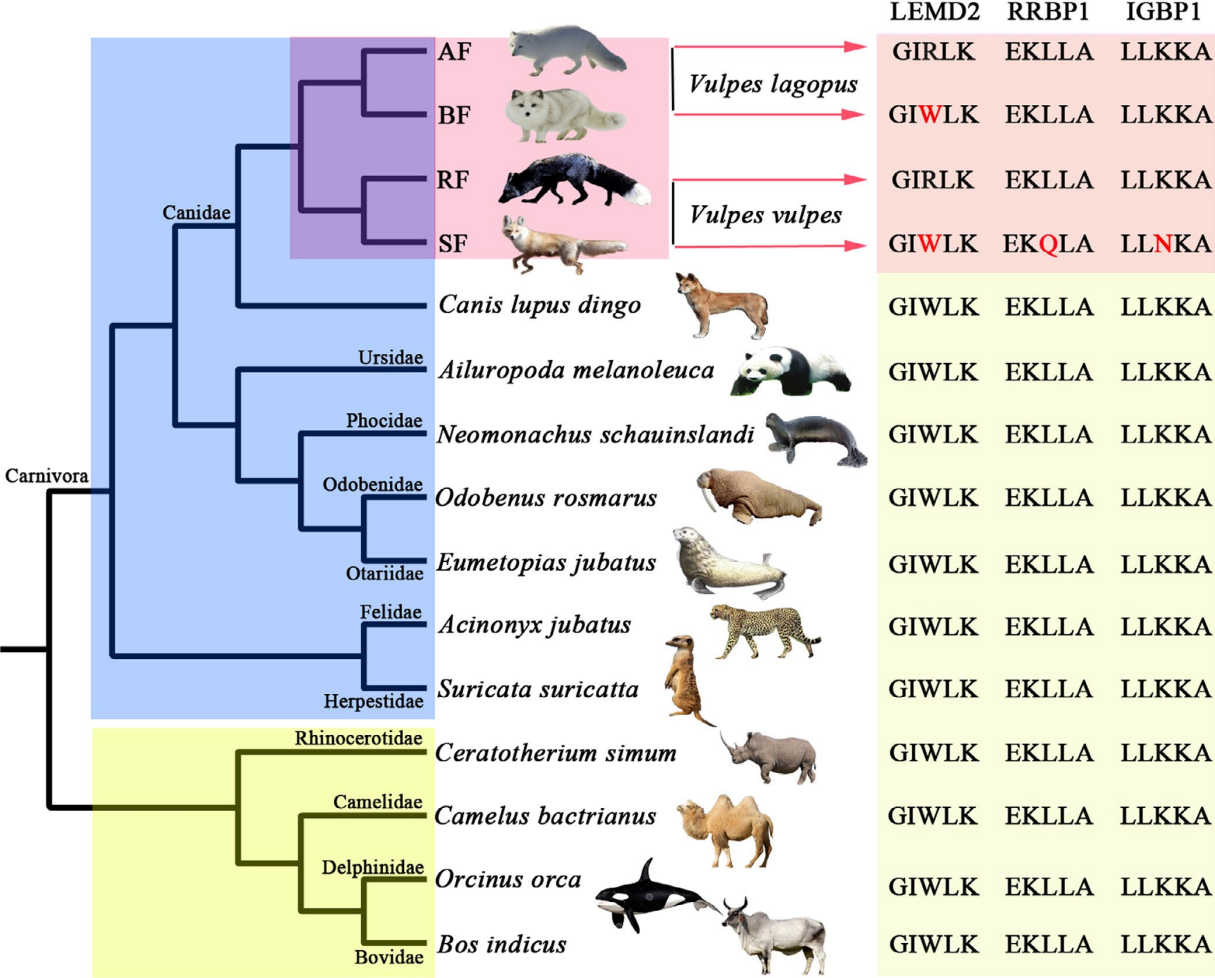
Specific mutation sites in the *RRBP1*, *IGBP1,* and *LEMD2* genes. Comparison of amino acid substitutions of three genes among the gene fragments of 11 Mammalia species. The shared mutation in the *LEMD2* gene is T235A, and the amino acid R occurs only in wild fox (arctic fox and red fox). The specific mutation in the *RRBP1* gene is A527T and amino acid Q occurs only in silver fox. The specific mutation in the *IGBP1* gene is G153C, and the amino acid N occurs only in silver fox

Specific mutations in 171 orthologous genes shared by two groups (AF vs. BF and RF vs. SF) were identified. According to these screening principles, three specifically mutated genes, *LEMD2*, *RRBP1,* and *IGBP1*, were identified. To verify the uniqueness of these mutations, the sequences of the three genes in 11 species covering the major mammalian lineages were downloaded from NCBI (Table S8). Among Carnivores (seven species), Artiodactyla (two species), Perissodactyla, and Cetacea, the specific mutations were found to occur exclusively in fox (Figure 4).

The *LEMD2* (LEM domain‐containing protein 2) gene in the wild foxes (AF and RF) was mutated from T to A at site 235, resulting in a change in the encoded amino acid from tryptophan (Trp, W) to arginine (Arg, R). The sequence alignment with the other 11 mammalian species also showed that this change occurred only in wild foxes (RF and AF). Moreover, the *RRBP1* (Ribosome binding protein 1) gene in the SF was mutated from A to T at site 527, resulting in a change in the encoded amino acid from leucine (Leu, L) to glutamine (Gln, Q), which was different from the sequence in RF. The *IGBP1* (Immunoglobulin binding protein 1) gene showed a patterns similar to that of *RRBP1*; in SF, it was mutated from G to C at site 153, resulting in a change in the encoded amino acid from lysine (Lys, K) to asparagine (Asn, N), and across the 11 species analyzed, this change occurred exclusively in SF.

## 4 DISCUSSION

Deciphering the genetic basis of animal domestication is an active research area. The availability of transcriptome sequences provides an efficient and economical opportunity to study this issue at the level of individual genes (Li et al., 2020). In our study, comparative transcriptome analysis of mixed RNA from three tissues (liver, brain, and kidney) was used to identify the selected genes and pathways related to the feeding adaptive evolution characteristics, such as changes in physiology, body size, energy metabolism and immunity, between wild and farmed foxes.

### 4.1 Positively selected genes might play a role in adaptation to strong seasonal fluctuations and energy requirements

Positive selection analyses identified eleven positively selected genes in AF versus BF and fourteen positively selected genes in RF and SF. Among those genes, several were related to protein anabolism (*GOLGA4*, *SLC35A2*, and *CEP19*), innate immunity (*CFI*, *LRRFIP1,* and *TMEM41B*) and DNA damage repair (*MDC1*). Of these, the protein encoded by the *CEP19* gene localizes mainly to centrosomes and primary cilia (Nishijima et al., 2017). Knockout experiments in mice showed that the deletion of the *CEP19* gene could lead to obesity in animals (Dayyeh et al., 2011; Adel et al., 2013). We speculate that this gene might play an important role in controlling obesity in fox. The protein encoded by the *CFI* gene is a complement regulator protein that may play a role in the immune system through the alterative pathway (AP) in the complement system in the absence of antibodies or the inability of antibodies to effectively bind. Current studies in several species (e.g., shark, rat, carp, and rainbow trout; Anastasiou et al., 2011; Nakao et al., 2003; Schlaf et al., 2010; Shin et al., 2009) show that this gene is conserved and is mainly expressed in the liver. In contrast to previous studies, this study detected the rapid evolution of this gene, suggesting that *CF1* may play an important role in the immune system of *Vulpes*. The *LRRFIP1* gene encodes a DNA recognition receptor that can recognize the transcription products or genomic DNA of pathogenic microorganisms entering the cell and plays a vital role in natural immunity (Yang et al., 2010). The *TMEM41B* gene encodes an evolutionarily conserved transmembrane protein. Previous studies have shown that this gene plays a crucial role in the normal neurotransmission of motor circuits in fruit flies and the normal development of motor axons in zebrafish embryos (Van Alstyne et al., 2018). Moreover, an early study showed that this gene regulates autophagy and that deletion of this gene inhibits autophagosome activity, in turn causing lysosomal damage (Moretti et al., 2018). The protein encoded by the *MDC1* gene is a DNA damage repair protein that can interact with phosphorylated histone *H2AX* at the DNA break point to promote the repair of DNA damage by ATM kinase and the meiosis recombination protein complex (Nabieh et al., 2009). In addition, this gene promotes the repair of DNA damage caused by ionizing radiation to maintain chromosome stability (Stewart et al., 2003).

Wild foxes (including the AF and RF) consume considerable energy to hunt and avoid predators, have high energy demands during the reproductive season (Prestrud et al., 1992), need to respond to starvation periods during winter, and react to extremely low temperatures through thermoregulation (Prestrud, 1991). As farmed foxes (including BF and SF) do not have to cope with seasonal fluctuations in food availability, this fluctuation is less pronounced in these species. Hence, these positively selected genes involved in protein synthesis, immunity, and DNA damage repair might play a role in adaptation to strong seasonal fluctuations and energy requirements as experienced by wild foxes. More interestingly, the selected genes vary between the two types of farmed foxes, suggesting that while common targets of selection related to domestication and improvement exist, different evolutionary solutions have arisen to achieve similar end‐points within these closely related domesticated species.

### 4.2 Specific gene mutations might be affected by artificial selection in farmed foxes

A total of 117 orthologous genes shared by the two groups were used to identify the gene mutations specific to farmed foxes. Sequence analysis of these genes showed that the *LEMD2*, *RRBP1,* and *IGBP1* genes might be affected by artificial selection in farmed foxes. The protein encoded by the *RRBP1* gene is a ribosomal binding protein located on the endoplasmic reticulum and is a key gene for ribosomal binding, transport, and secretion of newborn proteins in mammalian cells (Benyamini et al., 2009). Previous studies have shown that this gene can mediate the interaction between the endoplasmic reticulum and microtubules (Cui et al., 2012) and can bind to actin (Diefenbach et al., 2004). Christopher et al. showed that *RRBP1* can affect the stability and translation of collagen mRNA after muscle overload (Fry et al., 2017). This result indicated that the expression of this gene was of great significance for the control of muscle fiber hypertrophy in animals. The *LEMD2* gene is a nuclear membrane protein gene in the LEM domain. Knockout of this gene in mice caused a significant increase in the activity of MAP kinase and AKT kinase, resulting in embryo death (Brachner et al., 2005; Tapia et al., 2015). The *IGBP1* gene is named immunoglobulin binding protein 1 (Jiang et al., 2013; Kong et al., 2009). Previous studies have shown that the gene is associated with the regulation of protein phosphatase activity, activation of B cells, and apoptosis (Nieradka et al., 2007; Prickett & Brautigan, 2007). Mutation of this gene can lead to developmental delays in intelligence and behavior (Graham et al., 2003). Because of the pleiotropic nature of the gene, the effect of the other two gene‐specific mutations (*LEMD2* and *IGBP1*) on fox adaptation is unclear, and further functional studies are needed.

### 4.3 SSR screening provides reliable genetic markers for the selection of superior fox varieties

Fox is an important fur‐bearing animal, and its characteristics, especially fur‐related characteristics, have high economic value. The development of modern genetics and molecular biology has provided excellent methods and tools for further improving varieties of farmed fox. Polymorphic SSR markers play important roles in population genetics and genetic diversity research, comparative genomics, linkage mapping, and association analysis (Wei et al., 2011). In the present study, 157,759 (AF: 22,546, BF: 81,614, RF: 15,216, SF: 38,383) potential microsatellite sites were identified among all the fox transcriptome datasets. Agarose gel electrophoresis verification showed that 90% and 50% of primers were successful for amplification in BF and SF, respectively. The SSR markers developed in this study can be used to construct high‐resolution genetic linkage maps and to perform gene‐based phenotypic association analyses in fox. Moreover, the SSR dataset will provide new markers for future population genetic and species identification studies.

Although we used transcriptome data to screen out some positive selection genes and mutation sites that might be related to domestication, we had to face the limitations of the transcriptome and the small sample size. We plan to improve these questions by increasing the sample size and using genomic data in the following studies.

## 5 CONCLUSIONS

Understanding the genetic changes that underlie genetic variation in fox may expand our understanding of the genetic modifications that are enriched upon domestication by humans. This study represents the first comparative transcriptome analysis of wild and farmed fox and reveals a number of candidate genes that may be involved in phenotypic differences in foxes in domesticated versus wild environments. Protein synthesis and DNA damage repair genes were under positive selection in farmed species, and the three genes with specific mutations screened from farmed species might play a role in adaptation to strong seasonal fluctuations and energy expenditure as experienced by wild foxes. Furthermore, a large number of microsatellite markers were discovered and verified, providing new markers for the construction of high‐resolution genetic linkage maps and for gene‐based association analyses in foxes.

## CONFLICT OF INTEREST

The authors declare that they have no conflicts of interests.

## AUTHOR CONTRIBUTIONS

**Xiufeng Yang:** Conceptualization (equal); Formal analysis (lead); Software (lead); Writing‐original draft (equal). **Guangshuai Liu:** Formal analysis (equal); Funding acquisition (equal); Writing‐original draft (equal); Writing‐review & editing (lead). **Qi Wang:** Resources (equal). **Xiaodong Gao:** Resources (equal). **Tian Xia:** Resources (equal). **Chao Zhao:** Formal analysis (equal); Funding acquisition (equal). **Huashan Dou:** Resources (lead). **Honghai Zhang:** Conceptualization (equal); Funding acquisition (lead).

## ETHICAL APPROVAL

All sample procedures and experimental methods were approved by the Qufu Normal University Institutional Animal Care and Use Committee (Permit Number: QFNU2018‐013).

## Supporting information

Figure S1

Figure S2

Figure S3

Figure S4

Table S1

Table S2

Table S3

Table S4

Table S5

Table S6

Table S7

Table S8

## Data Availability

The raw RNA‐sequencing data are deposited in the Sequence Read Archive (SRA) database with accession number of SAMN15015145, SAMN15015146 and SAMN15015147 (Blue fox) and SAMN15015148 and SAMN15015149 (Silver fox).
