# Supplementary figures and images for: Comparative transcriptome provides insights into the selection adaptation between wild and farmed foxes
Source: Ecol Evol. 2021 Aug 30;11(19):13475–86. doi: 10.1002/ece3.8071 (PMC8495804; doi:10.1002/ece3.8071)

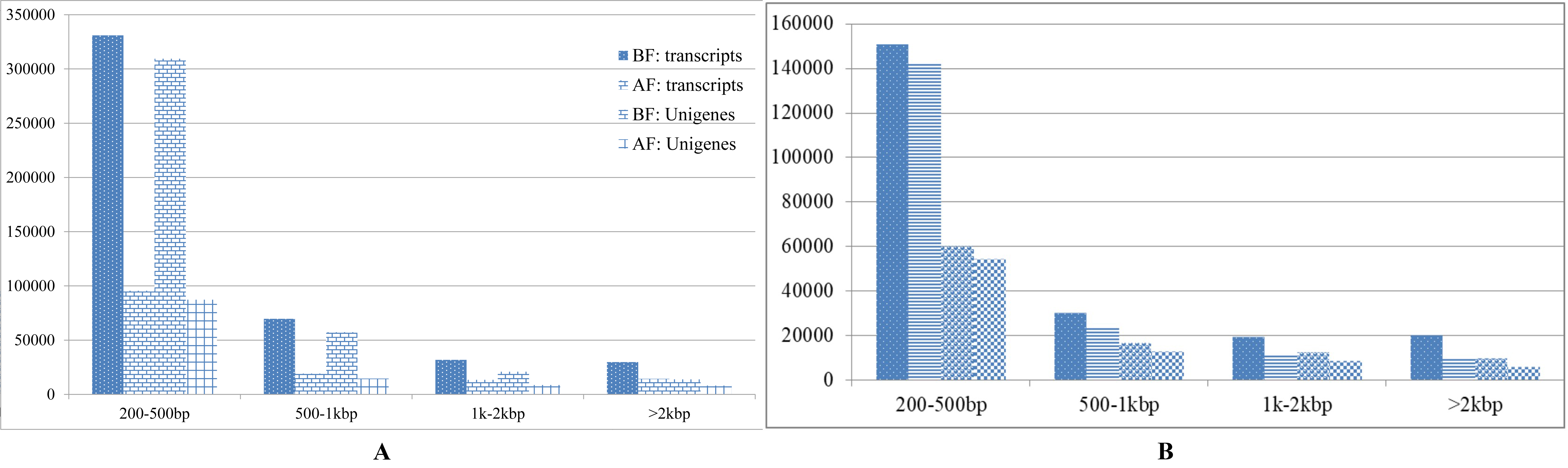

Supplement: Supplementary file 1 — Figure S1 [file ECE3-11-13475-s009.jpg]

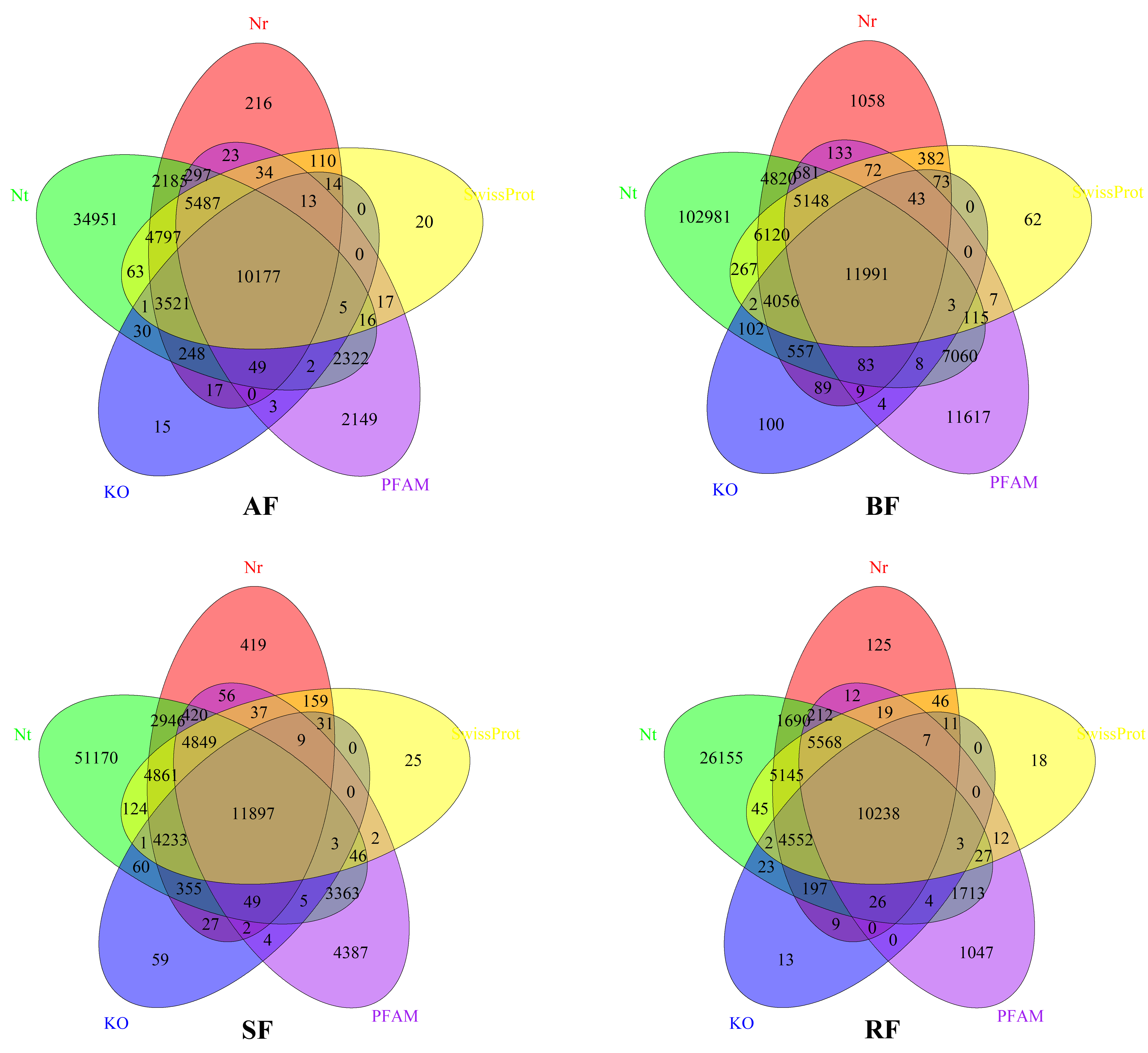

Supplement: Supplementary file 2 — Figure S2 [file ECE3-11-13475-s008.jpg]

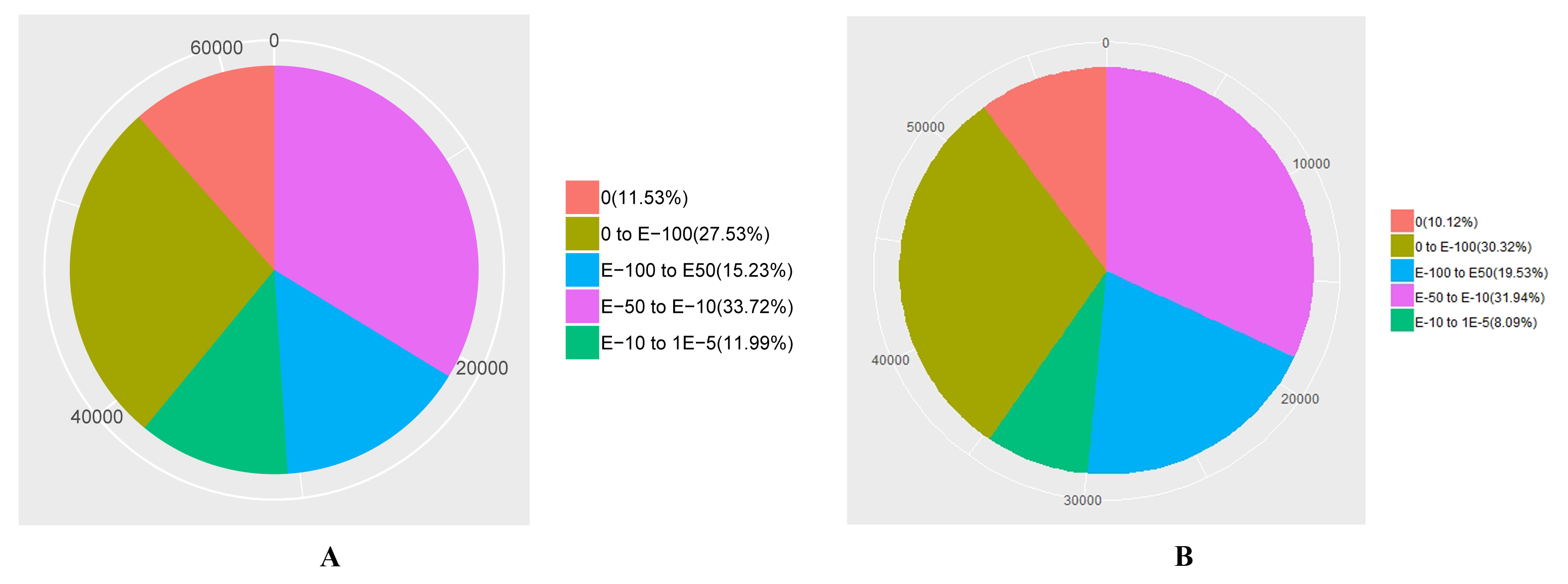

Supplement: Supplementary file 3 — Figure S3 [file ECE3-11-13475-s003.jpg]

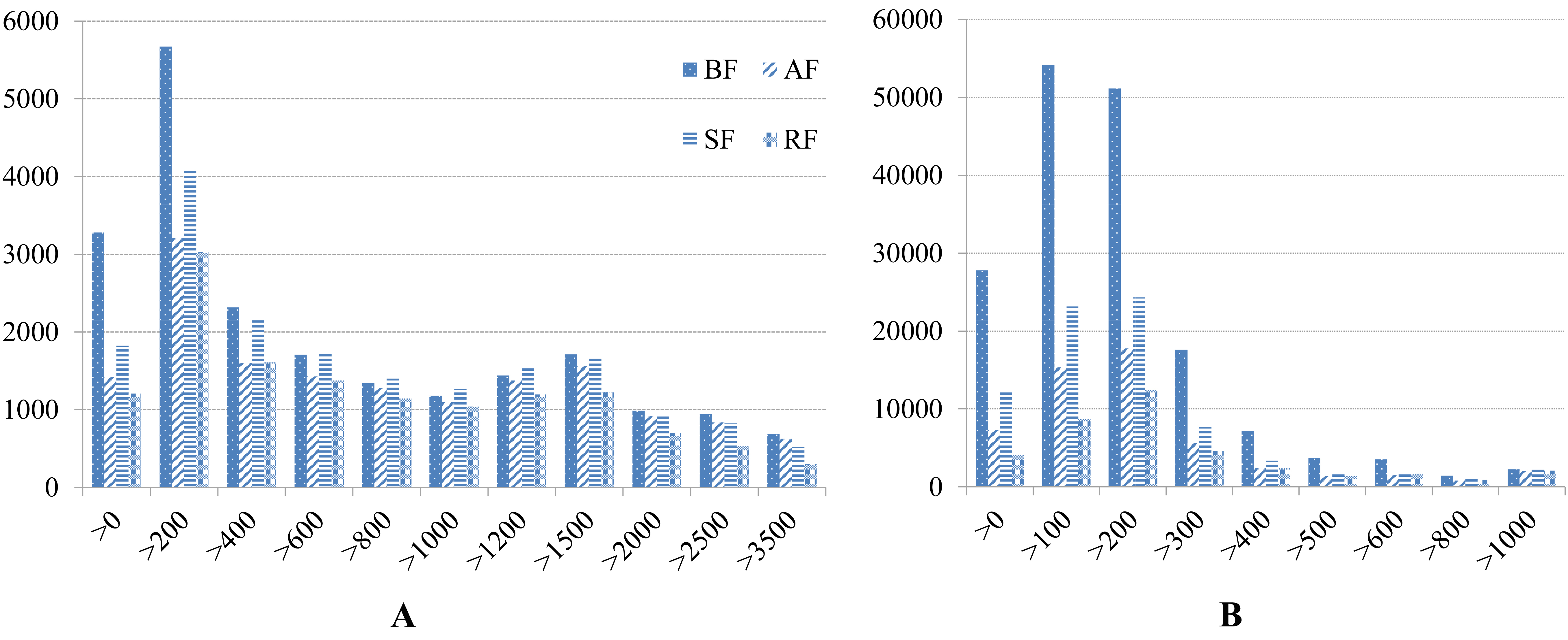

Supplement: Supplementary file 4 — Figure S4 [file ECE3-11-13475-s007.jpg]
