# Supplementary material for: Comparative transcriptome provides insights into the selection adaptation between wild and farmed foxes
Source: Ecol Evol. 2021 Aug 30;11(19):13475–86. doi: 10.1002/ece3.8071 (PMC8495804; doi:10.1002/ece3.8071)
Supplement: Supplementary file 5 — Table S1 [file ECE3-11-13475-s002.docx]

**Supplementary Table 1** The website and parameters of database.

| Database | Website | Parameter |
| --- | --- | --- |
| Nt | http://www.ncbi.nlm.nih.gov/ | NCBI blast 2.2.28+ |
|  |  | e-value = 1e-5 |
| Nr | http://www.ncbi.nlm.nih.gov/ | diamond v0.8.22 |
|  |  | e-value = 1e-5, --more-sensitive |
| KEGG | http://www.genome.jp/kegg/ | KAAS，KEGG Automatic Annotation Server  e-value= 1e-10 |
| Swiss-Prot | http://www.ebi.ac.uk/uniprot/ | diamond v0.8.22 |
|  |  | e-value = 1e-5, --more-sensitive |
| PFAM | http://pfam.sanger.ac.uk/ | HMMER 3.0 package，hmmscan |
|  |  | e-value = 0.01 |
| GO | http://www.geneontology.org/ | e-value = 1e-6 |
| KOG/COG | http://www.ncbi.nlm.nih.gov/COG/ | diamond v0.8.22 |
|  |  | e-value = 1e-3, --more-sensitive |
