# Supplementary material for: Comparative transcriptome provides insights into the selection adaptation between wild and farmed foxes
Source: Ecol Evol. 2021 Aug 30;11(19):13475–86. doi: 10.1002/ece3.8071 (PMC8495804; doi:10.1002/ece3.8071)
Supplement: Supplementary file 6 — Table S2 [file ECE3-11-13475-s012.docx]

**Supplementary Table** **2** PCR reaction system and condition.

| Reagent | Volume（μl） | Temperature | Time |  |
| --- | --- | --- | --- | --- |
| DNA | 3 | 94℃ | 10min |  |
| Forward Primer | 0.6 | 94℃ | 30s | 35 Cycle |
| Reverse Primer | 0.6 | Tm | 30s |  |
| 10 x PCR Buffer（Mg^2+^ plus） | 2.5 | 72℃ | 30s |  |
| BSA | 0.15 | 72℃ | 10nin |  |
| dNTP | 2 |  |  |  |
| Taq | 0.15 |  |  |  |
| water | 16 |  |  |  |
| Total | 25 |  |  |  |
