# Supplementary material for: Comparative transcriptome provides insights into the selection adaptation between wild and farmed foxes
Source: Ecol Evol. 2021 Aug 30;11(19):13475–86. doi: 10.1002/ece3.8071 (PMC8495804; doi:10.1002/ece3.8071)
Supplement: Supplementary file 7 — Table S3 [file ECE3-11-13475-s011.docx]

**Supplementary Table 3** Length distribution of Contigs and Unigenes.

| Sample | Transcript length interval（Number/Percent） | | | | Total Number | Unigenes length interval（Number/Percent） | | | | Total Number |
| --- | --- | --- | --- | --- | --- | --- | --- | --- | --- | --- |
|  | 200-500bp | 500-1kbp | 1k-2kbp | >2kbp |  | 200-500bp | 500-1kbp | 1k-2kbp | >2kbp |  |
| AF | 95499 67.1% | 19194 13.5% | 13434 9.4% | 14230 10.0% | 142357 | 87276 73.6% | 14543 12.3% | 8687 7.3% | 8071 6.8% | 118577 |
| BF | 330923 71.6% | 69624 15.1% | 31849 6.9% | 29796 6.4% | 462192 | 309451 77.1% | 57342 14.3% | 20918 5.2% | 13809 3.4% | 401520 |
| RF | 59813 61.3% | 16335 16.7% | 12064 12.4% | 9404 9.6% | 97616 | 53885 67.4% | 12395 15.5% | 8012 10.0% | 5608 7.0% | 79900 |
| SF | 150865 68.7% | 29851 13.6% | 18967 8.6% | 19879 9.1% | 219562 | 142188 76.0% | 23502 12.6% | 11470 6.1% | 9828 5.3% | 186988 |
