# Supplementary material for: Comparative transcriptome provides insights into the selection adaptation between wild and farmed foxes
Source: Ecol Evol. 2021 Aug 30;11(19):13475–86. doi: 10.1002/ece3.8071 (PMC8495804; doi:10.1002/ece3.8071)
Supplement: Supplementary file 8 — Table S4 [file ECE3-11-13475-s006.docx]

**Supplementary Table 4** Statistics of blast results for Unigenes against databases.

| Annotation database | BF | | AF | | SF | | RF | |
| --- | --- | --- | --- | --- | --- | --- | --- | --- |
|  | Unigene Nums | Percentage（%） | Unigene Nums | Percentage（%） | Unigene Nums | Percentage（%） | Unigene Nums | Percentage（%） |
| Annotated in Nr | 35315 | 8.79 | 27188 | 22.92 | 30350 | 16.23 | 27857 | 34.86 |
| Annotated in Nt | 143994 | 35.86 | 64151 | 54.1 | 84382 | 45.12 | 55600 | 69.59 |
| Annotated in KO | 17120 | 4.26 | 14095 | 11.88 | 16735 | 8.95 | 15085 | 18.88 |
| Annotated in SwissProt | 28,341 | 7.05 | 24,275 | 20.47 | 26,277 | 14.05 | 25,693 | 32.15 |
| Annotated in PFAM | 36,974 | 9.2 | 20,594 | 17.36 | 25,129 | 13.43 | 18,888 | 23.64 |
| Annotated in GO | 37,211 | 9.26 | 20,752 | 17.5 | 25,326 | 13.54 | 19,065 | 23.86 |
| Annotated in KOG | 9,129 | 2.27 | 8,908 | 7.51 | 9,245 | 4.94 | 9,305 | 11.64 |
| Annotated in all Databases | 6,007 | 1.49 | 5,324 | 4.48 | 5,996 | 3.2 | 5,314 | 6.65 |
| Annotated in at least one Database | 157,643 | 39.26 | 66,782 | 56.31 | 89,599 | 47.91 | 56,919 | 71.23 |
| Total Unigenes | 401,520 | 100 | 118,577 | 100 | 186,988 | 100 | 79,900 | 100 |
