# Supplementary material for: Comparative transcriptome provides insights into the selection adaptation between wild and farmed foxes
Source: Ecol Evol. 2021 Aug 30;11(19):13475–86. doi: 10.1002/ece3.8071 (PMC8495804; doi:10.1002/ece3.8071)
Supplement: Supplementary file 9 — Table S5 [file ECE3-11-13475-s010.docx]

**Supplementary Table 5** Blast results of Unigenes against databases.

| Sample | 0 | 0 to E-100 | E-100 to E-50 | E-50 to E-10 | E-10 to 1E-5 | Total |
| --- | --- | --- | --- | --- | --- | --- |
| AF | 3789 | 8780 | 4621 | 12624 | 5501 | 35315 |
| BF | 3417 | 8428 | 4898 | 8453 | 1992 | 27188 |
| SF | 3383 | 9127 | 5315 | 9205 | 3320 | 30350 |
| RF | 2508 | 8519 | 6054 | 9387 | 1389 | 27957 |
