# Supplementary material for: Comparative transcriptome provides insights into the selection adaptation between wild and farmed foxes
Source: Ecol Evol. 2021 Aug 30;11(19):13475–86. doi: 10.1002/ece3.8071 (PMC8495804; doi:10.1002/ece3.8071)
Supplement: Supplementary file 10 — Table S6 [file ECE3-11-13475-s004.docx]

**Supplementary Table** **6** The statistical results of SSR.

| Item | Number | | | |
| --- | --- | --- | --- | --- |
|  | BF | AF | SF | RF |
| Total number of identified SSRs | 81614 | 22546 | 38383 | 15216 |
| Number of SSR containing sequences | 66091 | 17573 | 29935 | 12137 |
| Total number of sequences examined | 401520 | 118577 | 186988 | 79900 |
| Total size of examined sequences (bp) | 209148339 | 74566356 | 107231995 | 53304583 |
| Number of sequences containing more than 1 SSR | 11543 | 3698 | 6165 | 2367 |
| Number of SSRs present in compound formation | 3331 | 1018 | 1827 | 654 |
| Mono-nucletide | 58820（72.1%） | 16282（72.2%） | 26299（68.5%） | 11050（72.6%） |
| Di-nucletide | 13540（16.6%） | 3626（16.1%） | 6262（16.3%） | 2364（15.5%） |
| Tri-nucletide | 7161（8.8%） | 2189（9.7%） | 4832（12.6%） | 1495（9.8%） |
| Tetra-nucletide | 2035（2.5%） | 422（1.9%） | 945（2.5%） | 282（1.9%） |
| Penta-nucletide | 32 | 17 | 22 | 14 |
| Hexa-nucletide | 26 | 10 | 23 | 11 |
