# Supplementary material for: Comparative transcriptome provides insights into the selection adaptation between wild and farmed foxes
Source: Ecol Evol. 2021 Aug 30;11(19):13475–86. doi: 10.1002/ece3.8071 (PMC8495804; doi:10.1002/ece3.8071)
Supplement: Supplementary file 11 — Table S7 [file ECE3-11-13475-s001.docx]

**Supplementary Table** **7** SSR primer and result for PCR amplification.

|  | Gene ID | Type | Forward Primer | Reverse Primer | Size | S/F |
| --- | --- | --- | --- | --- | --- | --- |
| AF Vs BF | c168462_g1 | (TG)7 | AGGAGCCTGAGTTCTGTCCA | TCTGGGCCAAAGGTACTGAT | 176 | S |
|  | c326078_g1 | (GT)7 | CAGACTGCCTCTGGTCTGTG | TCTCATCTGAAGGGCCCAGA | 136 | S |
|  | c381697_g1 | (TTA)5 | AGCAACAGTCTTCCACACTT | ACCACATCAAAGTGCTGGGT | 103 | S |
|  | c155352_g4 | (GCC)6 | AGGTTTGAGCGGCTGATCTC | CGTGTTGTTGTTGCTGCTGA | 258 | S |
|  | c99965_g1 | (TGTC)5 | ACCTGGCTGAGCTCAGTTTC | TCTCCTACCATGTGCCCTGA | 251 | S |
|  | c167474_g1 | (AATG)5 | TCCACGAGAGCTGAGATCCT | GCTTTAGTTTGCGGTGCCAT | 239 | S |
|  | c150624_g1 | (TCAAT)6 | TGTCCCTTTCTGGTAGTGCT | AGCTCCCTAAGGACAAGATCA | 138 | S |
|  | c163593_g1 | (AGCCCG)6 | CTCGGCGAGCACCAGGAG | GGAAGTCGGCCAGCACTT | 268 | S |
|  | c122384_g1 | (GCACGA)5 | GGACCGGAGTATCAGAAGCG | CTCCTCCTCCTCGGCAGAG | 240 | F |
|  | c166847_g1 | (TTTCCT)15 | GTGCCAGGTTCTCATTCCCA | TGAGGAAAACCGCCAAGGAA | 146 | S |
| RF vsSF | c39032_g1 | (TC)8 | TCAGTCAGTTAGGCAGCTGC | ACTCAAAATCTGGAGCCTGCA | 227 | F |
|  | c134996_g1 | (AG)6 | GATCGCCATTATCCCCAGCA | CCTGATCTAGGGCTGAGACCT | 170 | S |
|  | c38968_g1 | (GCC)5 | TGCACTTCCAGCTTCTGAGG | TCCATCCTGTCTTGCTGAACA | 270 | F |
|  | c57731_g2 | (CGC)5 | GGAGGTTCAGGCTTTGTGGA | GGGACCCACCTGGAAGCG | 234 | F |
|  | c59905_g6 | (TGGA)5 | TTCACCCCTAGGAGACCTGG | TCATTCACCATGTCCCTGCC | 188 | S |
|  | c61666_g2 | (TCTT)5 | AAGTTGCCTGCAGATAGGGC | TCTCTACCATCCAGCACCCT | 180 | S |
|  | c54957_g1 | (CGGCC)5 | GGCCTCACTGATGCACTGTT | GGAGAACCTGAAGCCCCAAG | 273 | F |
|  | c47874_g1 | (CCACA)5 | TACACTCACAACCGAGCCAC | TCCACCATCCTGTCTTGTGC | 191 | S |
|  | c57617_g2 | (GCTCCC)10 | CTCCCGCTCCTGCTCCAG | CTCCTGGCCCACGTGGAT | 238 | F |
|  | c47088_g1 | (GGAGCA)5 | CAGCACCCTCCAGGAGAAAG | CCATGGACAGCTCAGCTCTC | 114 | S |

Note：S/F: S:Successful, F:Faild.
