# Supplementary material for: Comparative transcriptome provides insights into the selection adaptation between wild and farmed foxes
Source: Ecol Evol. 2021 Aug 30;11(19):13475–86. doi: 10.1002/ece3.8071 (PMC8495804; doi:10.1002/ece3.8071)
Supplement: Supplementary file 12 — Table S8 [file ECE3-11-13475-s005.docx]

**Supplementary Table** **8** The data download from NCBI.

| Gene | Scientific Name | Accession Number | Gene | Scientific Name | Accession Number |
| --- | --- | --- | --- | --- | --- |
| *RRBP1* | *Canis lupus dingo* | XM_025469236.1 | *LEMD2* | *Canis lupus dingo* | XM_025418460.1 |
|  | *Neomonachus schauinslandi* | XM_021700748.1 |  | *Neomonachus schauinslandi* | XM_021684581.1 |
|  | *Odobenus rosmarus divergens* | XM_004412293.2 |  | *Odobenus rosmarus divergens* | XM_004407719.2 |
|  | *Eumetopias jubatus* | XM_028112981.1 |  | *Eumetopias jubatus* | XM_028120561.1 |
|  | *Acinonyx jubatus* | XM_027069523.1 |  | *Acinonyx jubatus* | XM_027057969.1 |
|  | *Suricata suricatta* | XM_029917057.1 |  | *Suricata suricatta* | XM_029943159.1 |
|  | *Ceratotherium simum simum* | XM 014790065.1 |  | *Ceratotherium simum simum* | XM_004424470.2 |
|  | *Camelus bactrianus* | XM_010972901.1 |  | *Camelus bactrianus* | XM_010949065.1 |
|  | *Orcinus orca* | XM_033429362.1 |  | *Orcinus orca* | XM_004267720.2 |
|  | *Bos indicus* | XM_019973212.1 |  | *Bos indicus* | XM_019985959.1 |
| *IGBP1* | *Canis lupus dingo* | XM_025466092.1 |  |  |  |
|  | *Neomonachus schauinslandi* | XM_021680256.1 |  |  |  |
|  | *Odobenus rosmarus divergens* | XM_004396625.2 |  |  |  |
|  | *Eumetopias jubatus* | XM_028092094.1 |  |  |  |
|  | *Acinonyx jubatus* | XM_015080584.2 |  |  |  |
|  | *Suricata suricatta* | XM_029930415.1 |  |  |  |
|  | *Ceratotherium simum simum* | XM_004439880.2 |  |  |  |
|  | *Camelus bactrianus* | XM_010973630.1 |  |  |  |
|  | *Orcinus orca* | XM_004275783.1 |  |  |  |
|  | *Bos indicus* | XM_027533778.1 |  |  |  |
